# Tissue-Specific Approaches Reveal Diverse Metabolic Functions of Rho-Kinase 1

**DOI:** 10.3389/fendo.2020.622581

**Published:** 2021-02-09

**Authors:** Taylor Landry, Daniel Shookster, Hu Huang

**Affiliations:** ^1^ East Carolina Diabetes and Obesity Institute, East Carolina University, Greenville, NC, United States; ^2^ Department of Kinesiology, East Carolina University, Greenville, NC, United States; ^3^ Human Performance Laboratory, College of Human Performance and Health, East Carolina University, Greenville, NC, United States; ^4^ Department of Physiology, East Carolina University, Greenville, NC, United States

**Keywords:** Rho-kinase, metabolism, energy balance, glucose metabolism, lipid metabolism

## Abstract

Rho-kinase 1 (ROCK1) has been implicated in diverse metabolic functions throughout the body, with promising evidence identifying ROCK1 as a therapeutic target in diabetes and obesity. Considering these metabolic roles, several pharmacological inhibitors have been developed to elucidate the mechanisms underlying ROCK1 function. Y27632 and fasudil are two common ROCK1 inhibitors; however, they have varying non-specific selectivity to inhibit other AGC kinase subfamily members and whole-body pharmacological approaches lack tissue-specific insight. As a result, interpretation of studies with these inhibitors is difficult, and alternative approaches are needed to elucidate ROCK1’s tissue specific metabolic functions. Fortunately, recent technological advances utilizing molecular carriers or genetic manipulation have facilitated discovery of ROCK1’s tissue-specific mechanisms of action. In this article, we review the tissue-specific roles of ROCK1 in the regulation of energy balance and substrate utilization. We highlight prominent metabolic roles in liver, adipose, and skeletal muscle, in which ROCK1 regulates energy expenditure, glucose uptake, and lipid metabolism *via* inhibition of AMPK2α and paradoxical modulation of insulin signaling. Compared to ROCK1’s roles in peripheral tissues, we also describe contradictory functions of ROCK1 in the hypothalamus to increase energy expenditure and decrease food intake *via* leptin signaling. Furthermore, dysregulated ROCK1 activity in either of these tissues results in metabolic disease phenotypes. Overall, tissue-specific approaches have made great strides in deciphering the many critical metabolic functions of ROCK1 and, ultimately, may facilitate the development of novel treatments for metabolic disorders.

## Introduction

Rho-kinase (ROCK) belongs to the protein kinase A/G/C (AGC) subfamily of serine/threonine protein kinases and is a major downstream effector of small GTPase RhoA (1). The two ROCK isoforms, ROCK1 and ROCK2, each contain a kinase domain at its N-terminus, a central coiled-coil domain, and a pleckstrin-homology domain split by a cysteine-rich region at its C-terminus (2–4). ROCK1 and ROCK2 share 65% amino acid homology and have been implicated in a variety of cellular functions, including smooth-muscle contraction and actin cytoskeleton arrangement; however, these isoforms also perform independent functions due to differences in their structure, subcellular localization, and gene distribution (5, 6). For example, ROCK1 has uniquely been identified as an important regulator of energy balance and substrate metabolism. Pharmacological ROCK1 inhibitors, like Y-27632 and fasudil (HA1077), have been valuable to elucidating ROCK1’s diverse metabolic roles (7–12); however, both these compounds have varying selectivity to also inhibit ROCK2 and other AGC kinase subfamily members. The nonspecific and systemic effects of these inhibitors make interpretation of studies in which they are used challenging (13); therefore, alternative approaches to investigating ROCK1 function are required to understand its tissue-specific metabolic functions.

In this article, we review the physiological roles of ROCK1 in the regulation of energy balance and substrate utilization. We describe novel insights into ROCK1’s tissue-specific functions facilitated by recent technological advances and highlight prominent roles in liver, adipose, skeletal muscle, and hypothalamus. Furthermore, emerging evidence suggests ROCK1 is a molecular mediator underlying the pathogenesis of diabetes and obesity.

## ROCK1 in Liver

### Hepatic ROCK1 Overactivity Is Associated With Metabolic Disease States-

To date, the most well-documented metabolic roles of ROCK1 are observed in liver, with clear connections demonstrated between hepatic ROCK1 overactivity and humans or rodents with metabolic disorders (14–21). For example, in humans, liver ROCK1 protein content positively correlates with BMI, liver triglycerides (TG’s), and markers of liver damage including alanine transaminase and aspartate transaminase (14). Moreover, elevated hepatic ROCK1 activity has consistently been observed in a plethora of models of disordered metabolism including: humans with fatty liver disease (14), DIO mice (14), db/db mice (14), ob/ob mice (14), TNFα-treated hepatocytes (15), endothelial nitric oxide synthase (eNOS) deficient mice (16), palmitate-treated hepG2 cells (21), palmitate metabolite lysophosphatidylcholine (LPS) -treated Huh7 cells (20), LPS-treated mice (15), and DIO streptozotocin-treated rats (18). Consequently, ROCK1’s role in homeostatic and disordered liver metabolism has been an important subject of investigation.

The causal relationship between hepatic ROCK1 and metabolic disease has been investigated using a constitutively active ROCK1-specific mutant in the liver (L-CA-ROCK1), which increases ROCK1 activity 2-fold (14). In chow-fed L-CA-ROCK1 mice, body weight is normal, however fasting glucose levels and lipogenic gene expression including fatty acid synthase (FAS) and stearoyl-CoA desaturase (SCD1) is elevated. DIO L-CA-ROCK1 mice experience a more striking phenotype, characterized by accelerated obesity, insulin resistance, hepatic steatosis, hyperglycemia, and dyslipidemia. These mice also have decreased thermogenic gene expression indicated by decreased peroxisome proliferator-activated receptor gamma coactivator 1-alpha (PGC1α) and uncoupling protein 1 (UCP1) mRNA in brown adipose tissue (BAT) and/or white adipose tissue (WAT). Overall, the L-CA-ROCK1 phenotype demonstrates a possible causal role of hepatic ROCK1 overactivity in metabolic disease pathologies, identifying a potential therapeutic target in liver ROCK1 to treat obesity and diabetes.

### Inhibition of Liver ROCK1 Protects Against Metabolic Disease Pathologies-

Several studies have investigated the therapeutic potential of genetically or chemically inhibiting ROCK1 in various models of disordered liver metabolism (14–17, 20). One mouse model of liver ROCK1-deficiency (L-ROCK1^-/-^), in which hepatic ROCK1 activity was specifically knocked down 80%, resulted in significant protection from DIO and related comorbidities (14). In chow-fed L-ROCK1^-/-^ mice, there are no differences in body weight, body composition, or food intake; however, in DIO L-ROCK1^-/-^ mice, body weight and adiposity are reduced, at least in part, due to elevated energy expenditure and locomotor activity. Increases in energy expenditure may be due to augmented thermogenic gene expression in BAT (PGC1α, UCP1, COX7a1, COX8b, and ELOVL3) and WAT (COX8b) (14).

ROCK1 inhibition also improves insulin sensitivity, glucose clearance, fatty liver, and circulating lipid levels (14–17, 22). Chow-fed, DIO, and db/db L-ROCK1^-/-^ mice experience improved glucose clearance and insulin sensitivity, as well as decreased liver weight, TG’s, and cholesterol content (14). Supporting these findings, Y-27632 treatment in primary mouse hepatocytes abolishes TNFα-induced insulin resistance (15). L-ROCK1^-/-^ mice also have decreased lipogenic gene expression (FAS, SCD1, SREBP1c, and ELOVL2), despite no observed differences in gene expression involved glucose metabolism (14). Overall, studies have observed encouraging therapeutic potential of liver-specific ROCK1 inhibition in metabolic disease models, demonstrated by increased energy expenditure, improved insulin sensitivity, and attenuated lipid accumulation. These results underscore the value of determining the molecular mechanisms underlying ROCK1 function to further understand the pathology of diabetes and obesity.

### Hepatic ROCK1 Negatively Regulates AMPK Activity-

The effects of ROCK1 inhibition on energy balance and lipid metabolism are abolished in AMPKα2^-/-^ mice, suggesting a mechanistic relationship between liver ROCK1 and AMPK in metabolic regulation (16, 17). Hepatic ROCK1 decreases phosphorylation (thr172) and activity of AMPK, which is the proposed mechanism through which ROCK1 increases gene expression and decreases phosphorylation of ACC^ser79^ and SREBP1^ser372^ to increase lipogenesis (14, 16, 17). Interestingly, therapeutic agents metformin and paeoniflorin target hepatic ROCK1/AMPK signaling to improve steatosis and dyslipidemia in DIO mice (14) and palmitate treated HepG2 cells, respectively (21). In summary, hepatic ROCK1 appears to have a prominent role in promoting lipogenesis *via* suppression of AMPK activity and subsequent elevations in AMPK’s downstream targets SREBP1c and ACC. At this time, the upstream molecular mediators of ROCK1 pathologies are less clear, but a recent study determining TNFα stimulates NF-κB to activate hepatic ROCK1 in primary hepatocytes and LPS-treated mice may suggest the involvement of inflammatory pathways.

Interestingly, the ability of hepatic ROCK1 inhibition to improve glucose metabolism was found to be AMPKα2 independent, suggesting an alternative, not yet discovered mechanism (14, 16, 17). The role of ROCK1 in the regulation of insulin signaling is complex and tissue specific, with some studies reporting ROCK1 directly reduces phosphorylation of the tyrosine^612^ residue (23, 24) and induces phosphorylation of the serine^632/635^ residues (25, 26) on insulin receptor substrate 1 (IRS1). Consequently, ROCK1 activity has been associated with impaired insulin signaling in smooth muscle (23, 27), fibroblasts (28), adipose tissue (24), heart (29, 30), and leukocytes (31). Conversely, some studies observe ROCK1 to facilitate glucose uptake in adipocytes (25, 32, 33) and skeletal muscle (25, 33–35). These convoluted results are most likely due to differences in experimental models and the tissue-specific differences in ROCK1-mediated regulation of insulin signaling should be considered when developing therapeutic agents.

## ROCK1 and Adipose Tissue

### Adipocyte-Specific ROCK1 Inhibition Is Therapeutic in Models of Metabolic Disease-

ROCK1 activity is elevated in the adipose tissue of DIO and db/db mice, and adipocyte-specific inhibition of ROCK1 rescues many metabolic disease pathologies (24, 36). While adipose-specific ROCK1 disruption by 50% has no obvious phenotype in healthy mice (24), DIO mice experience improved insulin sensitivity and glucose clearance, despite no changes in adipogenesis, energy balance, or inflammation (24). The benefits are even greater when adipocyte-specific ROCK1 activity is reduced by ~83% (36), resulting in attenuated HFD-induced weight gain and improved insulin sensitivity independent of body weight changes. Furthermore, fasting insulin, fasting glucose, FFA’s, adipocyte growth, and macrophage infiltration are all reduced. Despite this encouraging therapeutic potential, the mechanisms underlying ROCK1-mediated adipose pathologies remain relatively unexplored.

### ROCK1 Is Critical to Adipose Insulin Signaling-

Despite the glucose-lowering effects of ROCK1 inhibition in mouse models of metabolic disease (24, 36), ROCK1 inhibition in cultured adipocytes impairs insulin-stimulated glucose uptake (25, 32, 33). ROCK1 activity is critical to insulin-stimulated phosphorylation of IRS1^ser632/635^ and PI3kinase activity (25, 32). Interestingly, insulin directly stimulates rho membrane translocation *via* PI3kinase in adipocytes (37), and PI3kinase inhibition abolishes ROCK1-mediated glucose transport (33). This suggests a circuitous, poorly understood, regulatory mechanism of insulin signaling, in which ROCK1 is both downstream and upstream of PI3kinase. Overall, ROCK1’s role in insulin signaling is complex, and the opposing effects of adipocyte-specific ROCK1 inhibition in healthy vs. pathological models, indicates an importance of basal ROCK1 activity to glucose homeostasis but also implicates its overactivity in metabolic disease pathologies.

### Adipose ROCK1 Is Involved in Adipocyte Differentiation and Lipid Metabolism-

Studies utilizing primary human and rodent adipocytes have revealed ROCK1 also regulates adipocyte differentiation and storage. For example, silencing of the ROCK1 antagonist “deleted in liver cancer 1” in both white and brown cultured adipocytes, and subsequent overactivation of ROCK1, results in decreased adipocyte differentiation, lipid accumulation, and adipogenic gene expression (fatty acid binding protein 4;FABP4 and adiponectin) (38, 39). Additional impairments in thermogenic (UCP1 and ELOVL3) and mitochondrial (cox7a1 and cox5b) gene expression are observed in BAT, as well as reduced mitochondrial respiration (38). Increased ROCK1 activity in cultured human adipocytes has also been shown to impair lipolysis and reduce protein levels of phosphorylated hormone sensitive lipase^ser660^ and adipose TG lipase (40). Overall, while these studies lack in-depth mechanistic insight, their findings suggest adipose ROCK1 is a physiological negative regulator of adipogenesis, lipolysis, and thermogenesis.

## ROCK1 and Skeletal Muscle Metabolism

### Skeletal Muscle ROCK1 Overactivity Is Associated With Metabolic Disease States-

Similar to adipose and liver, skeletal muscle ROCK1 expression and activity are elevated in rodent models of metabolic disease (26, 41–43). Conversely, one study observed no differences in basal vastus lateralis (VL) ROCK1 protein expression or activity between obese and lean humans (35), highlighting the importance of considering potential differences between rodent and human ROCK1 function. Further supporting the hypothesis that overactive ROCK1 is involved in metabolic disease pathologies, at least in mice, constitutively active skeletal muscle-specific ROCK1 (SM-CA-ROCK1) results in early-onset obesity, even when eating a normal diet (44). These mice exhibit reduced physical activity, decreased energy expenditure, impaired glucose clearance and insulin sensitivity, elevated fasting TG’s and cholesterol, and increased respiratory exchange ratio suggesting decreased fat utilization. They also experience decreased thermogenic gene expression in BAT and WAT, as well as reduced mitochondrial size and content specifically in Type I muscle fibers. Lastly, myogenic gene expression is altered in these mice including reduced irisin and IL13 mRNA by 60% and 25%, respectively.

### Skeletal Muscle ROCK1 Paradoxically Regulates Insulin Signaling-

The metabolic dysfunctions of SM-CA-ROCK1 mice may be due to impaired insulin signaling (41, 42). Lipid-induced geranylgeranyl diphosphate synthase 1 (GGPPS), a branchpoint enzyme in the mevalonate pathway involved in cholesterol synthesis, activates RhoA/ROCK1 signaling in muscle, which then increases inhibitory phosphorylation of IRS1^ser307^ to inhibit downstream signaling (41). This phenomenon is rescued in muscle-specific GGPPS knockout mice, as is insulin sensitivity and glucose homeostasis (41). ROCK1 also activates phosphatase and tensin homolog to inhibit phosphorylation of AKT in cultured myotubes, providing another mechanism for ROCK1-mediated negative regulation of insulin signaling (43). Interestingly, in L6 myotubes, insulin inhibits ROCK1 to promote AMPK2α activity and subsequently inhibit the lipogenic transcription factor SREBP-1c (42). This suggests a mechanism in which insulin may inhibit ROCK1 activity to prevent inhibitory IRS1 phosphorylation and ultimately facilitate downstream insulin signaling.

The association between skeletal muscle ROCK1 and metabolic dysfunction has been well-documented in animal models; however, much like ROCK1 in adipose tissue, basal ROCK1 activity may be essential to skeletal muscle glucose uptake. In humans, VL ROCK1 activity positively correlates with glucose disposal in lean subjects, while insulin-stimulated ROCK1 activity is impaired in those with diabetes or obesity, possibly due to elevated levels of the ROCK1 antagonist RhoE (35). Furthermore, systemic ROCK1 knockout impairs skeletal muscle insulin signaling, and ROCK1 suppression in myoblasts blunts glucose uptake in a PI3kinase-dependent manner (25, 33, 34). Overall, ROCK1-modulated glucose uptake in skeletal muscle is similarly paradoxical to its role in adipose tissue, both regarding mechanisms and complexity (Section 3.2) (25, 33).

## Summary of ROCK1 in Peripheral Tissues

**Figure 1:**
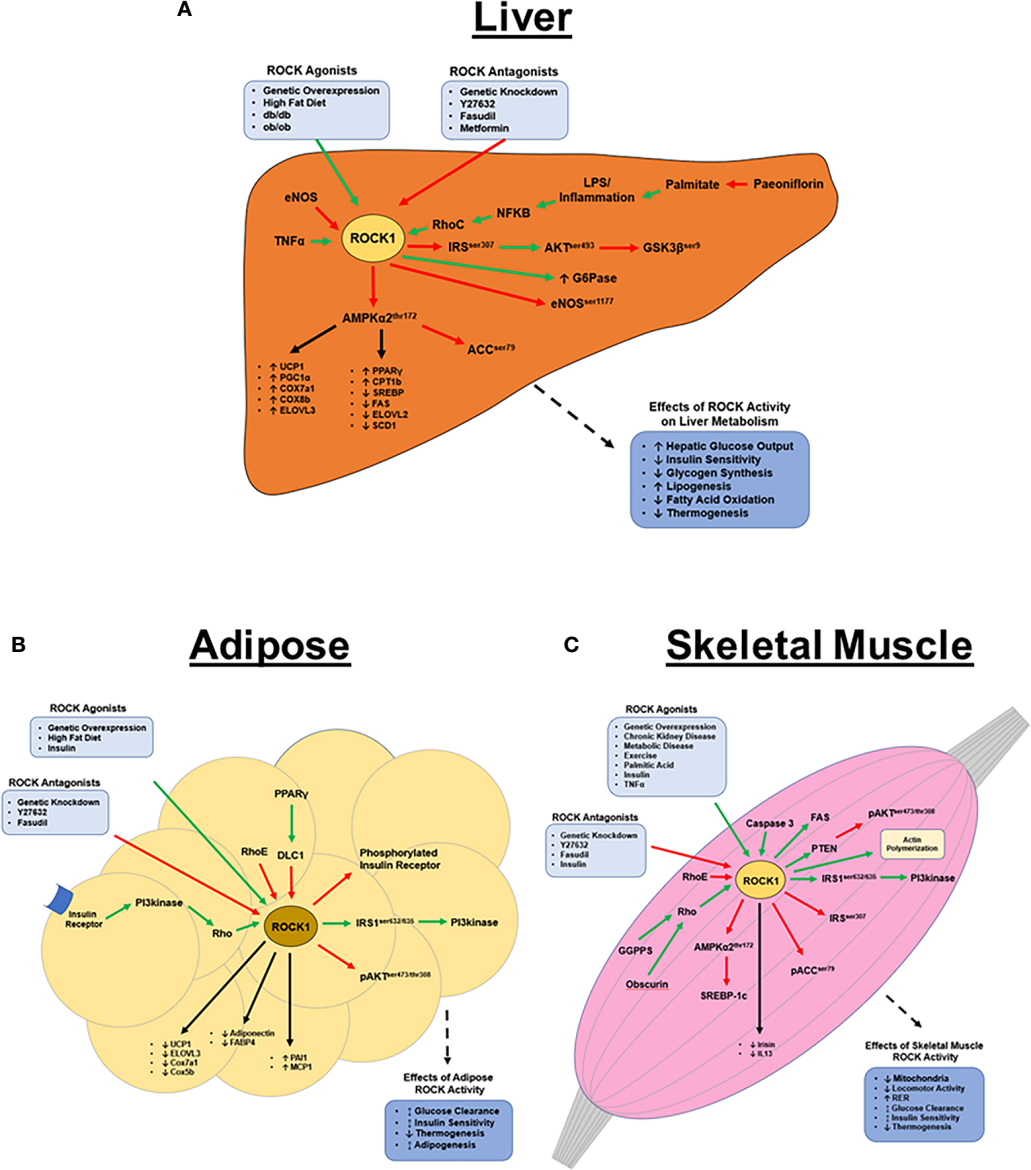
ROCK1’s metabolic functions in peripheral tissues. **(A)** Hepatic ROCK1 overactivity is associated with disordered metabolic regulation; conversely, downregulation of ROCK1 is therapeutic in metabolic disease models. ROCK1 primarily regulates lipid metabolism and thermogenesis *via* AMPK signaling, however the mechanisms underlying ROCK1’s role in insulin signaling remain unclear. The pathophysiology of ROCK1 overactivity in metabolic disease is poorly understood, however sustained inflammation and subsequent NF-κB signaling may be an upstream ROCK1 agonist. **(B)** Basal adipose ROCK1 activity is critical to homeostatic glucose metabolism; however, overactivity of ROCK1 is associated with metabolic disease phenotypes. ROCK1 is a negative regulator of thermogenic, adipogenic, and mitochondrial gene expression. Mechanistically, insulin and PI3kinase signaling are upstream activators of ROCK, while RhoE and DLC1 are antagonists. Downstream of ROCK1 includes a paradoxical insulin signaling mechanism, where ROCK1 activates IRS1 and PI3kinase, but also attenuates activation of insulin receptor and AKT. **(C)** Similar to in adipose, ROCK1 paradoxically regulates glucose metabolism in skeletal muscle. Basal ROCK1 function is critical to glucose regulation, but overactivity is associated with metabolic disease. ROCK1 regulates metabolism in skeletal muscle by downregulating AMPK, ACC, and AKT signaling, but also activates PI3kinase, FAS, and SREBP-1c. (Green arrows indicate activation; red arrows indicate inhibition; black arrows indicate regulation of gene expression).

To date, similar metabolic roles of ROCK1 have been identified in liver, skeletal muscle, and adipose tissue. ROCK1 in peripheral tissues inhibits AMPK2α, which results in changes in gene expression and downstream phosphorylation events to ultimately decrease energy expenditure and increase lipogenesis. ROCK1 also interferes with insulin signaling to increase blood glucose levels and ROCK1 overactivity is associated with metabolic disease states and related comorbidities including obesity, insulin resistance, and dyslipidemia. Despite this, increasing evidence suggests basal ROCK1 activity is also paradoxically essential to glucose disposal and insulin/PI3kinase signaling (**Figure 1**). Overall homeostatic ROCK1 function in peripheral tissues appears to be critical to metabolic health and future studies should focus on the differences between healthy and pathological ROCK1 activity.

## ROCK1 in the Central Nervous System (CNS)

### Hypothalamic ROCK1 Regulates Metabolism-

Unlike liver, adipose, and skeletal muscle, ROCK1 activity in the hypothalamus is reduced in db/db and DIO mice (45). Furthermore, hypothalamic ROCK1 knockout in healthy mice results in excessive food intake, dyslipidemia, and obesity, while ROCK1 overexpression has opposite effects (45, 46). One study observed fasudil treatment to increase food intake and gene expression of the orexigenic neuropeptide, neuropeptide Y, which is predominantly expressed in the arcuate nucleus of the hypothalamus (ARC) (47). Considering this, studies have identified novel roles for hypothalamic ROCK1 to regulate energy balance *via* ARC neuron populations.

### ROCK1 Regulates ARC Neurons-

The ARC, located in the medio-basal hypothalamus, contains both the orexigenic neuropeptide Y/agouti-related peptide (NPY/AgRP) -expressing and the anorexic proopiomelanocortin-expressing neuron populations (48–50). Disruption of ROCK1 in either of these neuron populations results in disordered neuronal activity and metabolism (45, 46). For example, deletion of ROCK1 in NPY/AgRP neurons results in increased NPY/AgRP activity and accelerated weight gain in chow-fed and DIO mice. These mice exhibit decreased resting energy expenditure and locomotor activity with increased serum TG’s (45, 46). Similarly, ROCK1 deletion in POMC neurons leads to POMC hypoactivity and obesity due to reduced locomotor activity, while whole-ARC ROCK1 deletion has even greater effects (46).

Tyrosine hydroxylase is the rate-limiting enzyme in dopamine synthesis (51), and activation of tyrosine hydroxylase-expressing (TH) neurons in the ARC has recently been shown to increase food intake and body weight (52). While RhoA deletion in TH neurons (RhoA-TH^-/-^) has no effects on energy balance in chow-fed mice, DIO RhoA-TH^-/-^ mice experience accelerated weight gain and adiposity due to increased food intake, despite no differences in energy expenditure or glucose regulation (53). Additionally, hypothalamic NPY and AgRP mRNA is elevated in RhoA-TH^-/-^ mice, suggesting RhoA/ROCK1 in TH neurons likely regulates other post-synaptic neuron populations as well (53). Overall, studies in the hypothalamus highlight a prominent role for ROCK1 to regulate ARC neurons involved in metabolic regulation; however, nonspecific ROCK1 knockdown in the hypothalamus results in a much more robust metabolic phenotype (45, 46, 53). Thus, ROCK1 likely regulates other, currently unidentified, neuron populations, in addition to NPY/AgRP, POMC, and TH neurons.

### ROCK1 Facilitates Hypothalamic Leptin Signaling-

Leptin is a potent adipokine that regulates ARC neurons to increase energy expenditure and suppress food intake (48, 53, 54). Deficiency in leptin, its receptor (LepR), or its downstream signaling results in hyperphagia, hyperglycemia, and obesity (48, 55). Interestingly, RhoA or ROCK1 deletion in NPY/AgRP, POMC, or TH neurons impairs leptin-mediated signaling and regulation of these respective neurons (45, 46, 53). Furthermore, hyper-leptinemia is observed in hypothalamic ROCK1 knockout mice, suggesting the involvement of ROCK1 in the development of leptin resistance seen in metabolic disease states (45, 46, 56).

Considering the strong association between ROCK1 and leptin activity, ROCK1 has been identified as a cell signaling molecule directly involved in LepR action. Following leptin binding to LepR, ROCK1 phosphorylates JAK2, which stimulates dimerization and phosphorylation of STAT3 (46). Phosphorylated STAT3 stimulates nuclear translocation and transcription of target genes, including POMC and signal of cytokine signaling 3 (SOCS3), each of which act to maintain energy homeostasis (57, 58). In addition to this leptin➔ROCK1➔JAK2➔STAT3 signaling mechanism, ROCK1 likely functions *via* other signaling pathways as well. For example, RhoA deletion in TH neurons also increases sensitivity to the hunger-inducing hormone ghrelin through unknown mechanisms (53). Insulin also modulates NPY/AgRP and POMC neurons and, like in peripheral tissues, may also facilitate hypothalamic ROCK1 function (54, 59–61).

### Summary of ROCK1 in the CNS-

**Figure 2:**
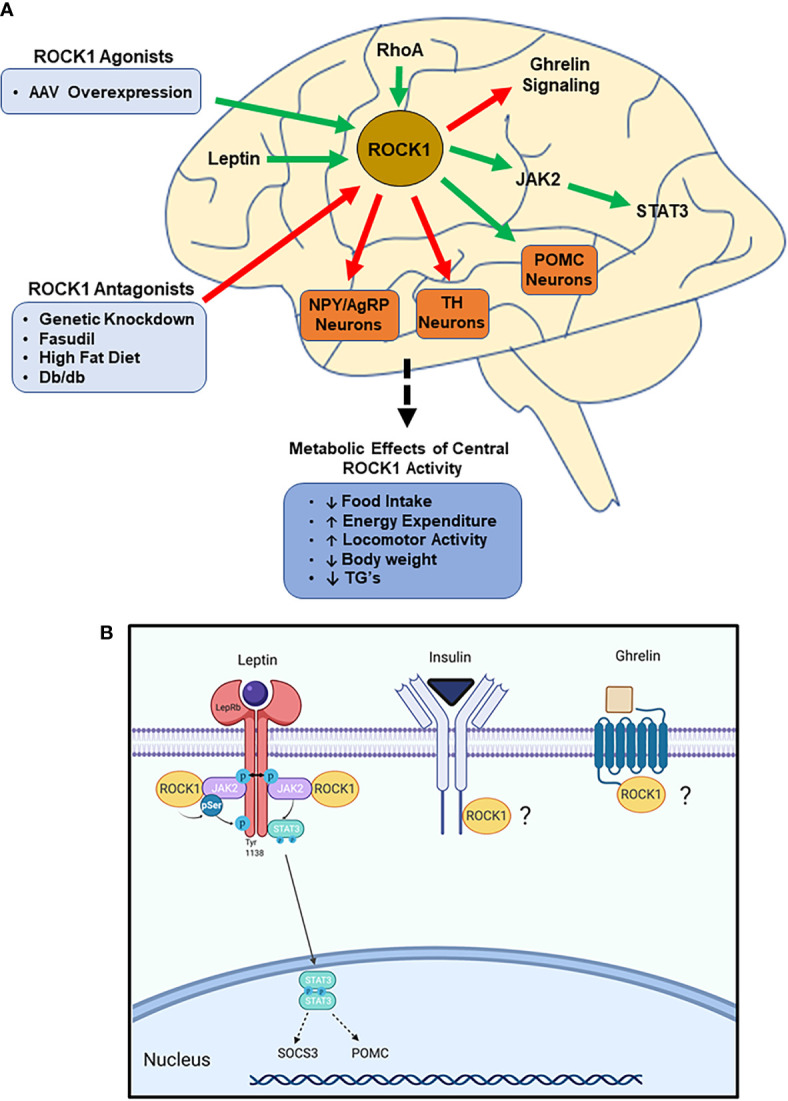
ROCK1 is critical to central nervous system (CNS)-mediated regulation of energy homeostasis. **(A)** Impaired ROCK1 activity in the hypothalamus is associated with disordered energy homeostasis. ROCK1 inhibits NPY/AgRP and TH neurons, while stimulating POMC neurons. Mechanistically, ROCK1 facilitates leptin signaling and attenuates ghrelin signaling. (Green arrows indicate activation; red arrows indicate inhibition). **(B)** Following leptin binding to LepR, ROCK1 phosphorylates JAK2, which stimulates dimerization and phosphorylation of STAT3. Subsequent STAT3 nuclear translocation elicits transcriptional changes including increased POMC and SOCS3 mRNA. Other potential mediators of hypothalamic ROCK1 action may be ghrelin and insulin receptor signaling.

In summary, hypothalamic ROCK1 regulates various neuron populations, including NPY/AgRP, POMC, and TH neurons, to decrease food intake and increase energy expenditure, with no obvious effects on glucose metabolism. Mechanistically, ROCK1 directly mediates leptin signaling and impairs ghrelin signaling through unknown mechanisms and the importance of these functions is underscored by obesity manifesting when hypothalamic ROCK1 function is impaired (**Figure 2**). The seemingly conflicting functions of central and peripheral ROCK1 are teleologically perplexing, and the reasons for these differences are unclear. Despite this. the various tissue-specific models described in this review cumulatively indicate both hypo- and hyper-ROCK1 activity have drastic metabolic effects, clearly demonstrating the critical nature of maintaining homeostatic ROCK1 function.

## Concluding Remarks

Many studies have used chemical inhibitors and whole-body genetic manipulation to identify ROCK1 as a prominent homeostatic regulator of diverse metabolic functions; however, these studies are limited in their isoform and tissue-specific insight. Recently, technological advances have facilitated development of novel models utilizing tissue-specific approaches, which have greatly enhanced our understanding of ROCK1’s functions. These studies have observed critical functions for ROCK1 in various metabolic tissues, including tissue-specific action in liver, adipose tissue, skeletal muscle, and hypothalamus to regulate food intake, thermogenesis, locomotor activity, glucose metabolism, and/or lipid metabolism. The molecular mechanisms underlying these functions are complex, underscored by disease states manifesting in response to ROCK1 overactivity, despite basal ROCK1 activity being critical to homeostatic maintenance of many physiological functions. Additionally, elevated ROCK1 activity consistently is associated with various metabolic disease states, suggesting ROCK1 may be useful as a preclinical marker of diabetes and obesity. Nonspecific ROCK1 inhibitors fasudil and Y-27632 demonstrate inhibitor pharmacotherapy is beneficial for these diseases; however, adverse effects such as hypotension, insulin resistance, and obesity are observed when ROCK expression/activity is non-specifically altered or systemically downregulated (25, 32–35, 62, 63). This once again highlights the importance of tissue-specific targeting of ROCK1, for example, *via* mannose-6-phosphate carriers (62, 64, 65), vitamin-A-coupled lysosomes (66), or genetic engineering. Overall, these tissue-specific approaches will greatly facilitate deciphering the many critical metabolic functions of ROCK1 and, ultimately, may result in the development of novel treatments for metabolic 2974disorders.

## Author Contributions

TL and DS wrote this manuscript. HH supervised and edited the manuscript. All authors contributed to the article and approved the submitted version.

## Conflict of Interest

The authors declare that the research was conducted in the absence of any commercial or financial relationships that could be construed as a potential conflict of interest.
